# Chemical-Genetic Interactions with the Proline Analog L-Azetidine-2-Carboxylic Acid in *Saccharomyces cerevisiae*

**DOI:** 10.1534/g3.120.401876

**Published:** 2020-10-20

**Authors:** Matthew D. Berg, Yanrui Zhu, Joshua Isaacson, Julie Genereaux, Raphaël Loll-Krippleber, Grant W. Brown, Christopher J. Brandl

**Affiliations:** * Department of Biochemistry, The University of Western Ontario, London, Canada; † Department of Biology, The University of Western Ontario, London, Canada; ‡ Donnelly Centre for Cellular and Biomolecular Research and Department of Biochemistry, University of Toronto, Toronto, Canada

**Keywords:** L-azetidine-2-carboxylic acid, proteotoxic stress, protein quality control, mistranslation, *Saccharomyces cerevisiae*, actin cytoskeleton

## Abstract

Non-proteinogenic amino acids, such as the proline analog L-azetidine-2-carboxylic acid (AZC), are detrimental to cells because they are mis-incorporated into proteins and lead to proteotoxic stress. Our goal was to identify genes that show chemical-genetic interactions with AZC in *Saccharomyces cerevisiae* and thus also potentially define the pathways cells use to cope with amino acid mis-incorporation. Screening the yeast deletion and temperature sensitive collections, we found 72 alleles with negative chemical-genetic interactions with AZC treatment and 12 alleles that suppress AZC toxicity. Many of the genes with negative chemical-genetic interactions are involved in protein quality control pathways through the proteasome. Genes involved in actin cytoskeleton organization and endocytosis also had negative chemical-genetic interactions with AZC. Related to this, the number of actin patches per cell increases upon AZC treatment. Many of the same cellular processes were identified to have interactions with proteotoxic stress caused by two other amino acid analogs, canavanine and thialysine, or a mistranslating tRNA variant that mis-incorporates serine at proline codons. Alleles that suppressed AZC-induced toxicity functioned through the amino acid sensing TOR pathway or controlled amino acid permeases required for AZC uptake. Further suggesting the potential of genetic changes to influence the cellular response to proteotoxic stress, overexpressing many of the genes that had a negative chemical-genetic interaction with AZC suppressed AZC toxicity.

Non-proteinogenic amino acids can be recognized by aminoacyl-tRNA synthetases, charged onto tRNAs and mis-incorporated into proteins [reviewed in Rodgers and Shiozawa (2008)]. L-azetidine-2-carboxylic acid (AZC) is a non-proteinogenic imino acid analog of proline produced by liliaceous plants as well as garden and sugar beets (Fowden 1959, 1972; Rubenstein *et al.* 2006). In species where AZC is not normally produced, it is activated by the prolyl-tRNA synthetase, charged onto tRNA^Pro^ and mis-incorporated into proteins at proline codons (Peterson and Fowden 1963). In nature, AZC inhibits growth of surrounding vegetation and poisons predators presumably by inducing protein mis-folding (Fowden and Richmond 1963). In mammals, AZC treatment leads to defects in the production of the proline rich protein collagen and results in limb deformation (Aydelotte and Kochhar 1972).

The proteotoxic stress that results from incorporating non-proteinogenic amino acids into proteins is similar to that arising from errors in protein synthesis during translation. Mistranslation occurs at a frequency of 10^−4^ to 10^−6^ (Joshi *et al.* 2019), and increases in response to different environmental conditions (Ling and Söll 2010; Wang and Pan 2016; Mohler *et al.* 2017) or due to mutations in the translational machinery (Moghal *et al.* 2014; Raina *et al.* 2014; Hoffman *et al.* 2017; Lant *et al.* 2017; Berg *et al.* 2017). Mis-incorporation frequencies as high as 8 to 10% can be achieved in yeast and *Escherichia coli* (Ruan *et al.* 2008; Mohler *et al.* 2017; Zimmerman *et al.* 2018; Berg *et al.* 2019).

The goal of our screen was to identify genes that show chemical-genetic interactions with AZC in *Saccharomyces cerevisiae* and thus systematically define the potential pathways cells use to cope with amino acid mis-incorporation. Using the yeast deletion (Giaever *et al.* 2002) and temperature sensitive collections (Costanzo *et al.* 2016), we identified 72 alleles with negative chemical-genetic interactions with AZC and 12 alleles that suppressed AZC-induced growth defects (positive chemical-genetic interaction). Genes encoding proteasomal proteins and proteins involved in endocytosis and actin cytoskeletal organization were identified as having negative chemical-genetic interactions, likely due to their being required for cells to tolerate AZC. These pathways were also required for tolerance to two other non-proteinogenic amino acids, canavanine, an arginine analog, and thialysine, a lysine analog, and to mistranslation resulting from a tRNA^Ser^ variant that mis-incorporates serine at proline codons. Genes with positive chemical-genetic interactions with AZC functioned mainly in the TOR signaling pathway and in controlling amino acid permeases.

## Materials And Methods

### Genetic screen for AZC interactions

The yeast deletion collection (Giaever *et al.* 2002) and temperature sensitive mutants (Ben-Aroya *et al.* 2008; Li *et al.* 2011; Kofoed *et al.* 2015; Costanzo *et al.* 2016; Berg *et al.* 2018) were arrayed in quadruplicate 1536 format with four replicate colonies for each mutant using a BioMatrix (S&P Robotics Inc.) automated pinning system on yeast extract-peptone medium with 2% glucose (YPD) containing 200 µg/mL geneticin (G418; Invitrogen). Newly pinned arrays were grown for 24 hr then used as the source to re-pin the cells first on synthetic defined (SD) medium containing uracil, leucine, histidine and methionine then on to the same medium containing 30 µg/mL AZC (ChemCruz). Cells were grown for 5 days at 30°. Plates were photographed every 24 hr after pinning.

### Calculating fitness in AZC and identification of hits

Fitness of each strain was defined as the ratio between colony size in medium containing AZC compared to colony size on medium lacking AZC. Raw colony size for each strain was determined using SGATools (Wagih *et al.* 2013). Further data analysis was performed using a custom R script (Supplemental File 1). Strains displaying small colony size in the absence of AZC, defined as growth less than 5% of the average colony size per plate, were removed from the analysis. Fitness of each strain was normalized using a Z-score method per plate. Briefly, the colony sizes were transformed so that the average colony size per plate was 0 and the standard deviation was set to 1. Strains with a Z-score greater than or equal to 3 were considered to have a positive chemical-genetic interaction; strains with Z-scores less than or equal to -1.5 were considered to have a negative synthetic interaction. Alleles of essential genes were grouped together and one Z-score calculated for the gene. Data can be found in supplemental file 2.

### Growth curve validation in AZC, canavanine and thialysine-containing media

Strains with negative and positive chemical-genetic interactions were validated in two independent growth curve experiments. In each experiment, strains were grown in biological triplicate overnight in YPD medium containing 200 µg/mL G418. Cells were washed with sterile water then resuspended in SD medium. Cultures were diluted to OD_600_ of 0.1 in either SD medium or SD medium containing 10 µg/mL AZC. Cells were incubated with agitation at 30° and OD_600_ measured every 15 min using a BioTek Epoch 2 microplate spectrophotometer for 24 hr. Area under the curve (AUC) was calculated for each strain using the R package ‘growthcurver’ (Sprouffske and Wagner 2016). The AUC values were used as a measure of fitness and normalized so that the wild-type strain in SD medium had a fitness of 1. The experimental fitness of each mutant grown in AZC medium (W_ij_) was compared to the expected fitness based on the fitness of the wild-type strain grown in AZC medium (W_i_) and the fitness of the mutant strain in SD medium (W_j_) using a multiplicative model (calculated as W*_ij_* – [W*_i_* * W*_j_*]). Strains in both replicate experiments that scored less than -0.1 (*P* ≤ 0.05; Welch’s *t*-test) were considered as having true negative chemical-genetic interactions while strains that scored greater than 0.1 (*P* ≤ 0.05; Welch’s *t*-test) were considered as having true positive chemical-genetic interactions. Data can be found in supplemental file 3.

Growth curves were performed as above in SD medium or SD medium containing 1.0 µg/mL L-canavanine (Sigma) or 7.5 µg/mL S-aminoethyl-L-cysteine (thialysine; Sigma). Interaction scores for the chemical-genetic interactions were calculated as above. Data can be found in supplemental file 4.

### Overexpressing genes with negative chemical-genetic interactions with AZC

Y6897 (*MATa **his3**Δ1 **leu2**Δ0 **ura3**Δ0 **met15**Δ0 **can1**Δ::**STE2**pr-S.p.HIS5 **lyp1**Δ::**STE3**pr-**LEU2*) containing genes with negative chemical-genetic interactions with AZC were obtained from the FLEX collection where each open reading frame is cloned on a *URA3* plasmid under a galactose inducible promoter (Hu *et al.* 2007; Douglas *et al.* 2012). Strains were grown in triplicate in media lacking uracil and containing raffinose as the carbon source. Cultures were diluted to OD_600_ of 0.1 in media lacking uracil and containing galactose either with 10 µg/mL AZC or without. AUC was calculated from 24 hr growth curves at 30° as above. The ratio of the AUC in media with and without AZC was calculated and compared to the control strain containing an empty plasmid. Strains with ratios twofold or greater that of the control strain and with a corrected *P*-value ≤ 0.05 were considered as suppressors of AZC induced toxicity. Data are found in supplemental file 6.

### Synthetic genetic array analysis of AZC hits with mistranslating tRNA^Ser^_UGG, G26A_

The SGA starter strain, Y7092 (*MATα **can1**∆::**STE2**pr-SpHIS5 **lyp1**∆ **his3**∆1 **leu2**∆0 **ura3**∆0 **met15**∆0*), was a kind gift from Dr. Brenda Andrews (University of Toronto). A wild-type tRNA^Ser^ or tRNA^Ser^_UGG, G26A_, a serine tRNA with a proline anticodon and a G26A mutation (Berg *et al.* 2017), were integrated at the *HO* locus with the *natNT2* cassette to create CY8611 (*MAT*α *HO**::natNT2 **can1**∆::**STE2**pr-SpHIS5 **lyp1**∆*) and CY8613 (*MAT*α *HO**::natNT2-**SUP17**_UGG, G26A_**can1**∆::**STE2**pr-SpHIS5 **lyp1**∆*) as described in Zhu *et al.* (2020).

For the SGA analysis, strains that showed negative synthetic interactions with AZC were re-arrayed in 384 format using the BioMatrix automated pinning robot. Three arrays were constructed. Each validated AZC sensitive allele was surrounded by a control *his3**Δ* strain to mitigate nutrient effects. The position of each AZC hit was randomized on each of the three arrays. The arrays were expanded to 1536-format where each strain was represented in quadruplicate on each plate. The SGA control and query strains (CY8611 and CY8613) were mated to each array at 22°. The SGA analysis was performed as described in Tong *et al.* (2001). Selected MAT*a* double mutants were grown at 30° for 5 days and imaged every 24 hr. Images from day 3 were analyzed and scored using SGAtools (Wagih *et al.* 2013) and a custom R script (supplemental file 1). Alleles with SGA score ≤ -0.1 and a corrected *P*-value ≤ 0.05 were called as synthetic with tRNA^Ser^_UGG, G26A_. Data can be found in supplemental file 5.

### Fluorescent microscopy and actin patch quantification

A wild-type strain was grown overnight in SD media and diluted to an OD_600_ of 0.1 in either SD media or SD media containing various concentrations of AZC (1 µg/mL, 2.5 µg/mL, 6 µg/mL, 10 µg/mL). Cells were grown to an OD_600_ of 1 before being fixed in 3.7% formaldehyde (Sigma). Cells were stained with fluorescein isothiocyanate-ConA, rhodamine-phalloidin, and 4’,6-diamidino-2-phenylindole as described in Ohya *et al.* (2005) and imaged with 100x magnification on a Zeiss Axio Imager Z1 Fluorescent microscope using ZEN Blue Pro software (Zeiss Inc.). The number of actin patches per cell was measured using CalMorph (v1.2; Ohya *et al.* 2005).

### GO term analysis, genetic interaction profiling and SAFE analysis

GO term analysis was performed using the GO term finder tool (http://go.princeton.edu/) using a *P*-value cut off of 0.01 after applying Bonferroni correction. Terms were filtered with REVIGO (Supek *et al.* 2011). GeneMANIA (Warde-Farley *et al.* 2010) was used to generate protein-protein and genetic interaction networks using the genetic interaction data from Costanzo *et al.* (2010, 2016) and all available protein-protein interactions (GeneMANIA datasets as of November 2019). Networks were constructed using Cytoscape 3.7 (Shannon *et al.* 2003). Spatial analysis of functional enrichment (SAFE; Baryshnikova 2016) analysis was performed through TheCellMap (http://thecellmap.org; Usaj *et al.* 2017).

### Data availability

Strains and plasmids are available upon request. The authors affirm that all data necessary for confirming the conclusions of the article are present within the article, figures, and tables. Supplemental data can be found on Figshare. Supplemental materials document contains all supplemental figures. Supplemental file 1 contains custom R script used to analyze the data. Supplemental file 2 contains the raw Z-scores from the initial AZC screen. Supplemental file 3 contains the AUC values and interaction scores for the AZC growth curve validation. Supplemental file 4 contains the AUC values and interaction scores for the canavanine and thialysine growth curves. Supplemental file 5 contain the results from the synthetic genetic array with AZC sensitive strains and the mistranslating tRNA^Ser^_UGG, G26A_. Supplemental file 6 contains AUC values from strains overexpressing genes with negative chemical-genetic interactions with AZC. Supplemental material available at figshare: https://doi.org/10.25387/g3.12783956.

## Results And Discussion

### A screen for genes that have chemical-genetic interactions with the proline analog AZC

**Figure 1:**
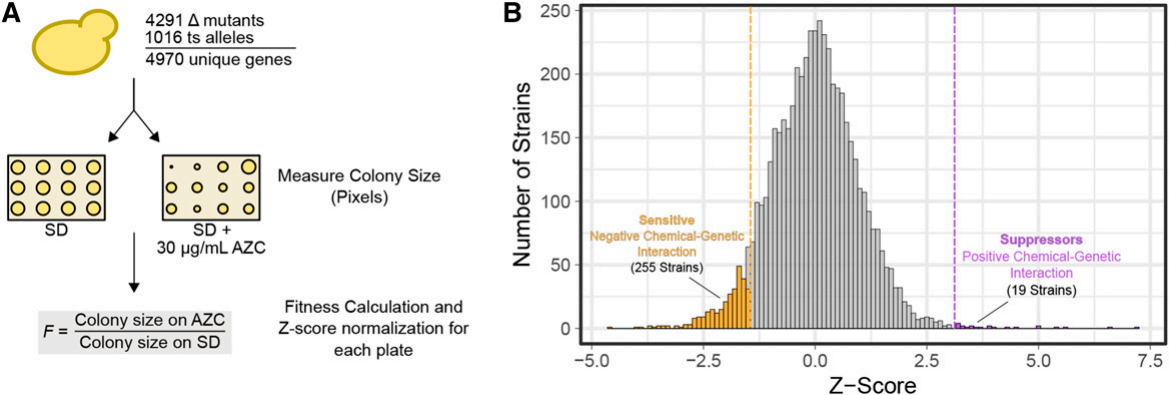
A screen identifying genes that have chemical-genetic interactions with AZC. (A) Overview of the screening approach. (B) Distribution of Z-score normalized fitness values for 5307 strains representing 4970 genes from the deletion and temperature sensitive yeast collections grown on medium containing 30 µg/mL AZC.

To identify genes and pathways that have chemical-genetic interactions with AZC, we measured the effect of AZC on growth of strains in the yeast deletion and temperature sensitive collections, containing 4291 and 1016 alleles respectively. Each strain in the collections was pinned in quadruplicate onto SD medium or SD medium containing 30 µg/mL AZC. We selected 30 µg/mL AZC as it decreased average fitness by ∼40% as measured by colony size (Figure S1). Fitness for each strain was determined from the ratio of the growth of each colony on medium with AZC to growth on medium without AZC after 5 days at 30°. Fitness values were normalized on a per plate basis and converted to Z-scores (Figure 1A). Nineteen genes (0.3% of the mutants screened) had a Z-score greater than 3, whereas 255 genes (5%) had a score less than -1.5 (Figure 1B; all raw colony sizes and fitness values are found in supplemental file 2). These strains were tested further for their potential suppression of AZC toxicity (positive chemical-genetic interaction) and for their sensitivity to AZC (negative chemical-genetic interactions), respectively.

The 255 strains with potential negative chemical-genetic interactions and the 19 strains with potential positive chemical-genetic interactions were analyzed for growth in liquid medium containing AZC. We tested different AZC concentrations with the wild-type strain and a potential AZC sensitive strain *hap5**Δ* (Figure S2). The *hap5**Δ* strain, with a Z-score of -3.5, was chosen as a representative AZC sensitive strain to ensure that the AZC concentration in the liquid growth assay allowed a dynamic range sufficient to detect differences in sensitivity of the other strains. An AZC concentration of 10 µg/mL was chosen for the validation as it decreased the fitness of the wild-type strain by ∼40% and the *hap5**Δ* strain by greater than 85%. The growth curve analysis in liquid medium confirmed a negative chemical-genetic interaction with AZC for 72 (28%) strains identified in the screen on solid medium: 43 from the deletion collection and 29 from the temperature sensitive collection (Supplemental File S3). This represents 1.0% and 2.8% of all deletion strains or temperature sensitive strains screened, respectively. The growth curve analysis validated 12 (63%) strains with positive chemical-genetic interactions; 6 from the deletion collection and 6 from the temperature sensitive collection.

### Defects in a variety of pathways result in negative chemical-genetic interaction with AZC

**Figure 2:**
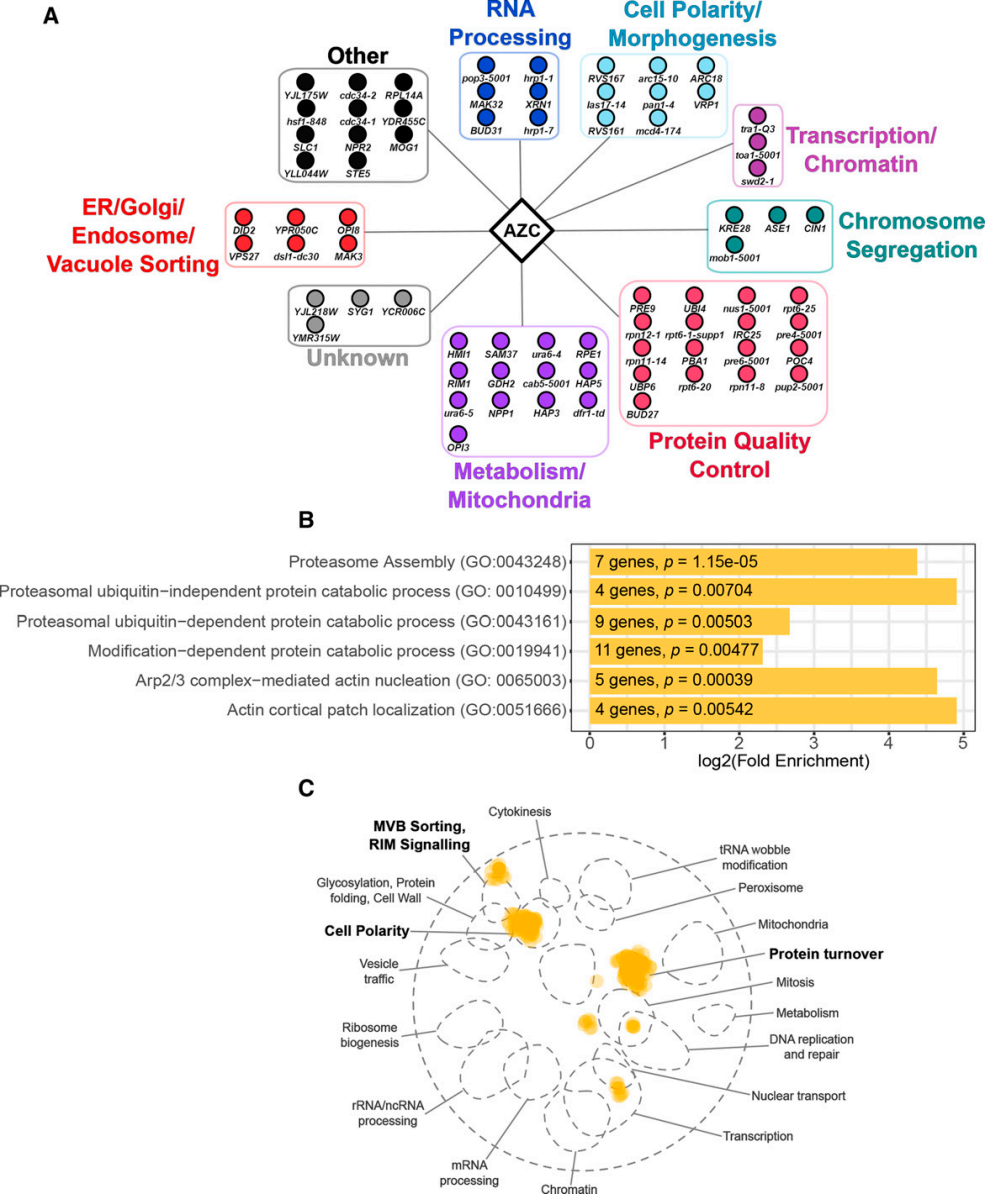
Negative chemical-genetic interactions with AZC. (A) Alleles that have negative chemical-genetic interactions with AZC are arranged according to their predicted function. (B) Significantly enriched GO biological processes were determined from the set of genes with negative chemical-genetic interactions with AZC. The number of genes identified corresponding to each GO term and their *P*-values are labeled on each bar. (C) SAFE analysis of genes that have negative chemical-genetic interactions with AZC were mapped onto the yeast genetic interaction profile map (Costanzo *et al.* 2016). Yellow dots represent genes within the local neighborhood of genes validated to have negative chemical-genetic interactions with AZC. Bold terms represent network regions that are enriched.

The gene functions of the 72 AZC strains with negative chemical-genetic interactions fell into 7 main categories when grouped based on their descriptions in the yeast genome database (www.yeastgenome.org; Figure 2A). Genes with a role in protein quality control were the most abundant (17 genes) followed by genes with a role in metabolism/mitochondria (13 genes). GO analysis of biological processes identified an enrichment of genes with roles in the proteasome and actin organization (Figure 2B). Spatial analysis of functional enrichment (SAFE), which detects network areas containing statistically overrepresented functional groups (Baryshnikova 2016), similarly showed enrichment in the protein turnover and cell polarity neighborhood of the yeast genetic interaction network (Figure 2C).

There are multiple mechanisms that could result in a negative chemical-genetic interaction with AZC. First, and most generally, nonredundant genes required for cellular response to proteotoxic stress will be identified since AZC is mis-incorporated into proteins at proline codons. Second, temperature sensitive alleles that are identified could be hypomorphs. Mis-incorporating AZC into the gene product would reduce the steady state level of the protein if the protein had one or more essential proline residues. Related to this, misincorporation into a hypomorphic protein could reduce function sufficiently to cause synthetic interactions with genes (either knockouts or temperature sensitive alleles) in secondary pathways. Third, AZC might act as a chemical inhibitor, independent of its misincorporation into proteins, specifically inhibiting a gene product. This would be similar, for example, to the inhibitory effect of aminotriazole on His3 (Klopotowski and Wiater 1965) and would result in a genetic interaction pattern closely resembling the gene whose protein is the target of AZC. Lastly, genes with negative chemical-genetic interactions with AZC could function to detoxify AZC, such as the AZC N-acetyltransferase *MPR1* found in AZC resistant Σ1278 yeast strains (Shichiri *et al.* 2001), or increase intracellular proline levels effectively diluting intracellular AZC concentrations (for example mutations in the γ-glutamyl kinase *PRO1*; Sekine *et al.* 2007).

**Figure 3:**
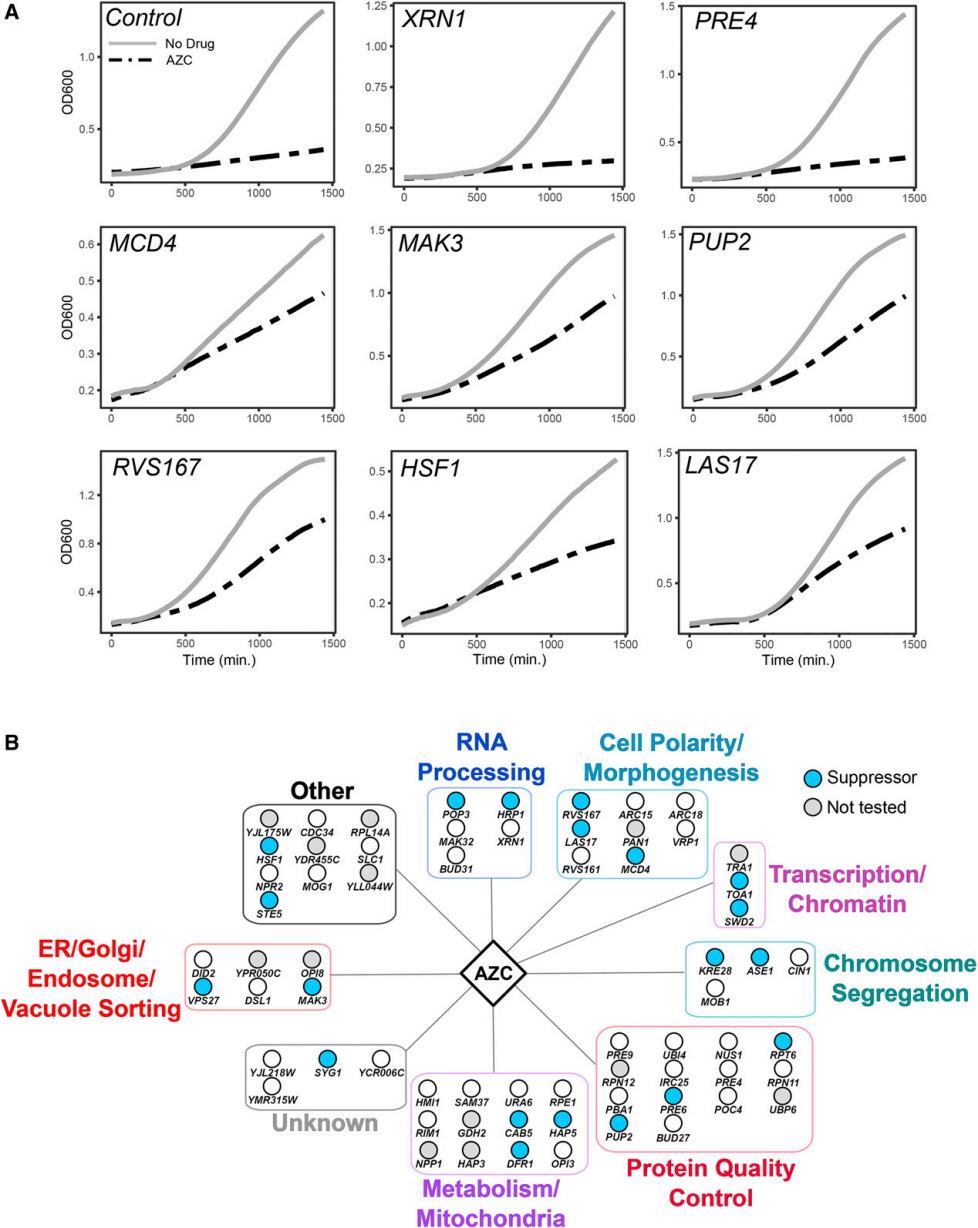
Overexpressing genes with negative chemical-genetic interactions with AZC can suppress AZC induced toxicity. Fifty-three of the genes having negative chemical-genetic interactions with AZC were obtained from the FLEX collection (Hu *et al.* 2007; Douglas *et al.* 2012). Overexpression strains and a control strain containing an empty plasmid were grown in medium lacking uracil and containing raffinose, diluted to an OD_600_ of 0.1 in medium lacking uracil containing galactose with or without 10 µg/mL AZC. Cells were grown for 24 hr at 30° and OD_600_ measured every 15 min. (A) Representative growth curves of the control strain, six strains that suppressed AZC toxicity (*MCD4**, **MAK3**, **PUP2**, **RVS167**, **HSF1* and *LAS17*) and two strains that did not suppress (*XRN1* and *PRE4*). (B) Area under the curve was calculated with the R package growthcurver (Sprouffske and Wagner 2016). Genes colored blue were considered to be suppressors of AZC toxicity when overexpressed and have a ratio of AUC plus/minus AZC twofold or greater than that of the control strain (*P ≤* 0.05).

We tested if genes having negative chemical-genetic interactions with AZC suppressed AZC induced toxicity when overexpressed by analyzing 53 strains found in the FLEX collection (Douglas *et al.* 2012). In the FLEX collection, individual genes are under *GAL_UAS_* control, being overexpressed in the presence of galactose. The 53 overexpression strains and a control strain containing an empty plasmid were grown in galactose-containing liquid medium with or without 10 µg/mL AZC and the ratio of the area under the curve was determined as a proxy of growth (Supplemental File 6). For the control strain, AZC reduced the AUC to 18.3% ± 7.5% of that in medium lacking AZC. Overexpressed genes that resulted in a twofold or greater ratio of AUC plus/minus AZC as compared to the control strain (*P* ≤ 0.05) were considered suppressors. Representative growth curves are shown in Figure 3A and S4. Figure 3A includes the control strain, two non-suppressor strains (*XRN1* and *PRE4*) and six suppressor strains. Twenty of the 53 genes suppressed AZC toxicity when overexpressed (Figure 3B). The overexpressed genes did not increase growth in medium lacking AZC, indicating that the suppression was related to AZC toxicity; however, overexpression of some of the genes (*e.g.*, *MCD4* and *HSF1*) reduced growth in the absence of AZC. The extent of suppression varied, being the greatest for *MCD4*, *MAK3* and *PUP2*, which increased AUC to 76%, 62% and 61% that seen in the absence of AZC, respectively. All of the functional groups contained one or more genes that suppressed, including three of the seven genes tested within the cell polarity/morphogenesis group and three of the 12 within the protein quality control group.

### Similar cellular processes are required for resistance to other amino acid analogs and tRNA-induced mistranslation

To evaluate if the negative chemical-genetic interactions identified here are specific for AZC, we determined the chemical-genetic interactions of the 72 AZC-sensitive alleles identified above with canavanine and thialysine, analogs of arginine and lysine, respectively, in liquid media.

**Figure 4:**
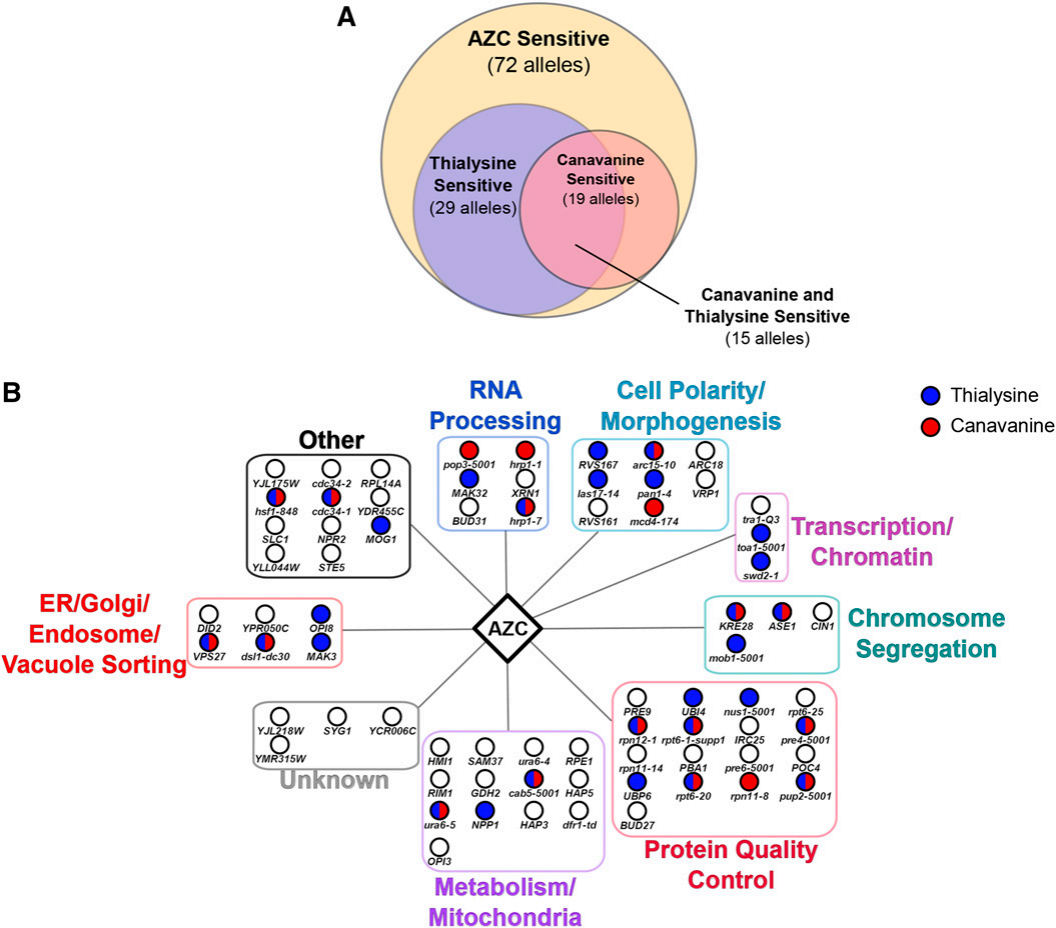
Negative chemical-genetic interactions with canavanine and thialysine for genes identified to have negative chemical-genetic interactions with AZC. (A) Venn diagram showing the overlap of genes with negative chemical-genetic interactions between AZC, canavanine and thialysine. (B) Alleles that were identified as having negative chemical-genetic interactions with AZC were tested for their chemical-genetic interactions with thialysine (blue) and canavanine (red) in liquid growth assays.

Both non-proteinogenic amino acids would give rise to proteotoxic stress, but their impact may vary depending upon the number and importance of arginine and lysine residues in specific proteins. From analyzing dose-response curves for each non-proteinogenic amino acid (Figure S3), we selected concentrations of 7.5 µg/mL for thialysine and 1.0 µg/mL for canavanine. These concentrations resulted in ∼40% decrease in wild-type growth, similar to the concentration used for AZC. Of the 72 alleles identified to have negative chemical-genetic interactions with AZC, 29 had a negative chemical-genetic interaction with thialysine and 19 with canavanine (Figure 4; See supplemental file S4 for interaction scores). Fifteen genes had a negative chemical-genetic interaction with all three amino acid analogs.

There were negative chemical-genetic interactions with canavanine and thialysine for genes in all of the functional groups with known functions. For thialysine, the greatest similarity with AZC was seen for genes with roles in ER/golgi/endosome/vacuole sorting (67% overlap), transcription/chromatin (67% overlap), cell polarity/morphogenesis (50% overlap). For canavanine, the greatest similarity with AZC was seen for genes with roles in RNA processing (50% overlap). Both thialysine and canavanine had negative chemical-genetic interactions with genes involved in protein quality control (47% overlap for thialysine and 35% overlap for canavanine). The negative chemical-genetic interaction of genes in all these pathways suggests that some genes in these pathways are required to respond to general amino acid mis-incorporation. We do note that although the pathways are similar for the three amino acid analogs, the specific genes identified were not identical. This could suggest that AZC mis-incorporation specifically and uniquely effects some genes in the identified pathways. Whether this represents absolute differences between the effects of AZC and other non-proteinogenic amino acids or differences in dose-response is unclear.

**Figure 5:**
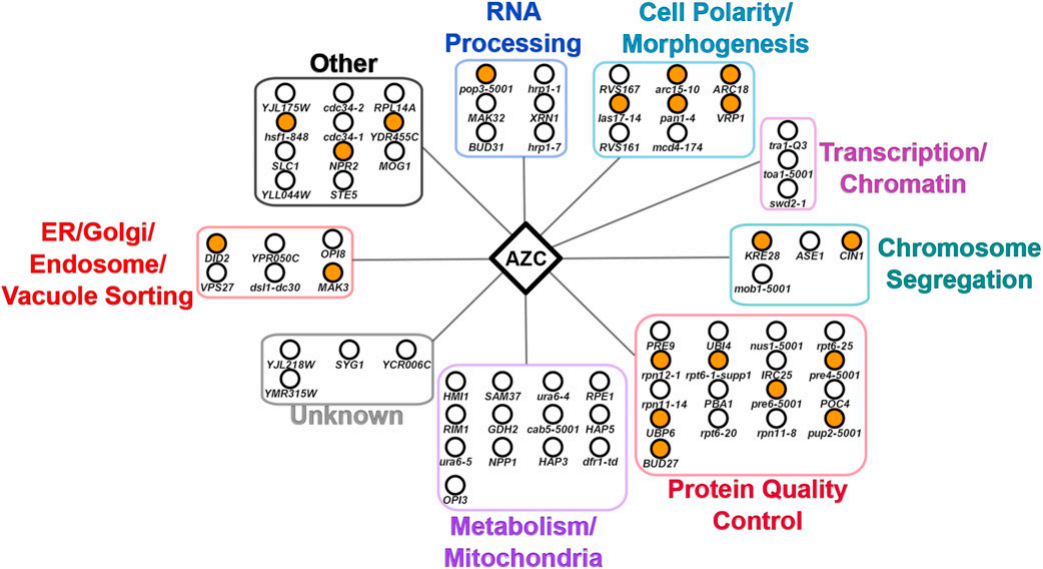
Negative genetic interactions between genes with negative chemical-genetic interactions with AZC and a tRNA that mistranslates serine at proline codons. Alleles that were identified as having negative chemical-genetic interactions with AZC were mated with a strain containing either a wild type tRNA^Ser^ or tRNA^Ser^_UGG, G26A_ which mistranslates serine at proline codons. Double mutants were selected using the SGA method described in Tong *et al.* (2001). Negative genetic interactions (orange) were determined by comparing double mutant fitness of the strains with tRNA^Ser^ to the strains with tRNA^Ser^_UGG, G26A_ in biological triplicate using SGAtools (Wagih *et al.* 2013) and a custom R script (supplemental file 1).

Next, we determined if alleles that have negative chemical-genetic interactions with AZC mis-incorporation for proline also have negative genetic interactions with expression of a tRNA that mis-incorporates a canonical amino acid in place of proline. This would provide evidence that native mistranslation provokes a similar response to the incorporation of a non-proteinogenic amino acid. Using a synthetic genetic array (Tong *et al.* 2001), we measured the fitness of double mutants containing one of the 72 alleles identified above in combination with a mistranslating tRNA^Ser^ variant with a UGG anticodon (Berg *et al.* 2017, 2019). This variant mis-incorporates serine at proline codons at ∼5% frequency and results in a 20% decrease in growth compared to a wild-type strain (Berg *et al.* 2019). Twenty of the 72 alleles tested had a negative genetic interaction with the mistranslating tRNA (Figure 5; See supplemental file S5 for interaction scores). The greatest overlap was seen for alleles with roles in cell polarity and morphogenesis (63%) and, as seen for canavanine and thialysine, protein quality control (41%). Genes in metabolism/mitochondria and transcription/chromatin did not have negative genetic interactions with tRNA derived mistranslation. It is possible the chemical-genetic interactions seen between the metabolism genes and the non-proteinogenic amino acids arise from nutrient signaling effects in response to these supplemented amino acids. It is also possible that tRNA^Ser^_UGG, G26A_ is not imported into the mitochondria, as there are two mitochondrial encoded tRNA^Ser^ genes in yeast (Turk *et al.* 2013). Whether the differences between tRNA derived mistranslation and AZC for genes in the other functional groups arise because of different impacts of AZC and serine on proteins or differences in dose-response is unclear, yet the overlap with AZC sensitive strains is evidence that many of the major pathways are shared.

### The proteasome is required to cope with proteotoxic stress caused by mis-incorporation

Twelve of the genes with negative chemical-genetic interactions with AZC encoded proteins in or involved in the assembly of the proteasome (*UBP6**, **RPT6**, **RPN11**, **PUP2**, **POC4**, **PRE6**, **PBA1**, **RPN11**, **PRE4**, **IRC25**, **RPN12**, **PRE9*). This supports previous reports demonstrating the requirement for proteasomal protein degradation for cells to cope with proteotoxic stress (Ruan *et al.* 2008; Hoffman *et al.* 2017; Shcherbakov *et al.* 2019). Consistent with this, the gene encoding ubiquitin (*UBI4*), a post-translational modification required in protein degradation signaling, also has a negative synthetic interaction with AZC and thialysine. These genes as well as *HSF1*, encoding the heat shock response transcription factor, almost certainly are required for cells to cope with proteotoxic stress. We note that no single gene encoding a heat shock chaperone was found to have a negative chemical-genetic interaction with AZC, likely due to genetic redundancy.

### The actin cytoskeleton has a role in protein quality control

**Figure 6:**
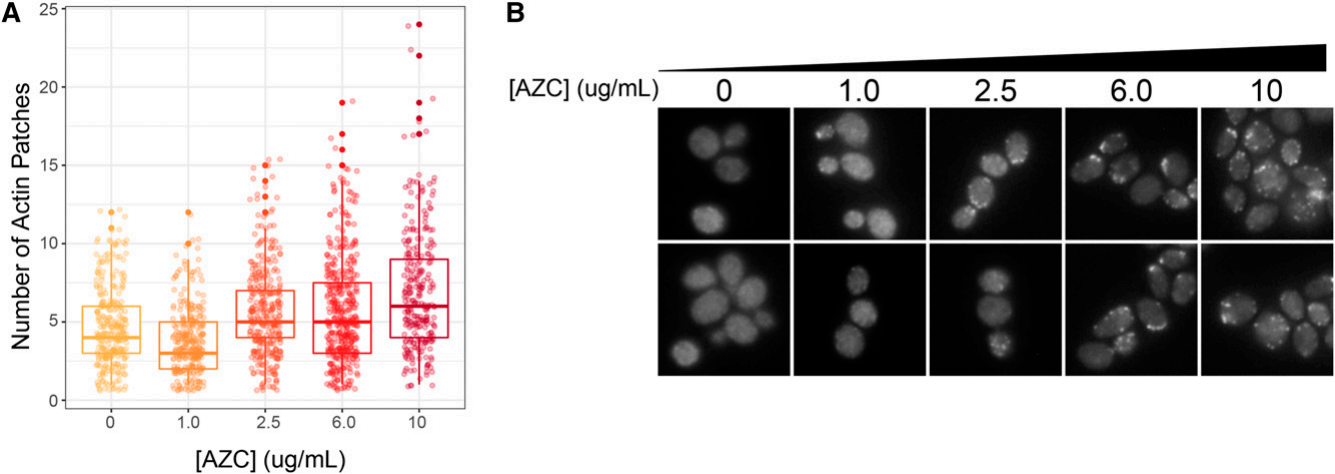
AZC treatment results in increased actin patches. (A) Cells were grown in various concentrations of AZC to an OD_600_ of 0.1, fixed, stained with fluorescein isothiocyanate-ConA, rhodamine-phalloidin, and 4’,6-diamidino-2-phenylindole as described in Ohya *et al.* (2005) and imaged with 100x magnification on a Zeiss Axio Imager Z1 Fluorescent microscope. Number of actin patches per cell was quantified using CalMorph (Ohya *et al.* 2005). In each condition, at least 270 cells were quantified. All conditions were statistically different from each other (*P* ≤ 0.05; Welch’s t-test) with the exception of the comparison between 2.5 µg/mL and 6 µg/mL AZC. (B) Representative images of cells quantified in (A).

In addition to genes involved in protein quality control, our screen identified other genes and pathways either required for resistance to mistranslation or particularly sensitive to mis-made protein. One of these additional pathways was actin patch organization and endocytosis. Interestingly, deletions or temperature sensitive alleles of genes involved in these pathways had a negative chemical-genetic interaction with all three amino acid analogs and tRNA derived mistranslation. These include two genes whose proteins are part of the ARP2/3 complex (*ARC15* and *ARC18*), which is required for motility and integrity of cortical actin patches (Winter *et al.* 1997) and five genes that regulate actin nucleation and endocytosis (*LAS17**, **VRP1**, **RVS161**, **RVS167* and *PAN1*; Naqvi *et al.* 1998; Duncan *et al.* 2001; Sun *et al.* 2006; Youn *et al.* 2010). To determine if AZC alters cellular actin, we imaged cells stained with rhodamine-labeled phalloidin to visualize actin after treatment with various AZC concentrations (Figure 6). The increase in number of actin patches per cell correlated directly with AZC concentration. In yeast, cortical actin patches play roles in endocytosis and cell wall remodelling. Negative genetic interactions were also observed between amino acid mis-incorporation and *vps27**Δ* and *did2**Δ*, two genes involved in vacuolar sorting (Piper *et al.* 1995; Nickerson *et al.* 2006). It is possible actin regulation and endocytosis pathways are required to turnover mis-made proteins at the plasma membrane. In support of this, Zhao *et al.* (2013) demonstrated that yeast cells defective in endocytic targeting and exposed to heat shock accumulate mis-folded cell surface protein and die due to loss of plasma membrane integrity. Amino acid mis-incorporation could have a similar effect on cell surface proteins.

### Genes involved in response to nitrogen compounds are required for AZC-induced toxicity

**Figure 7:**
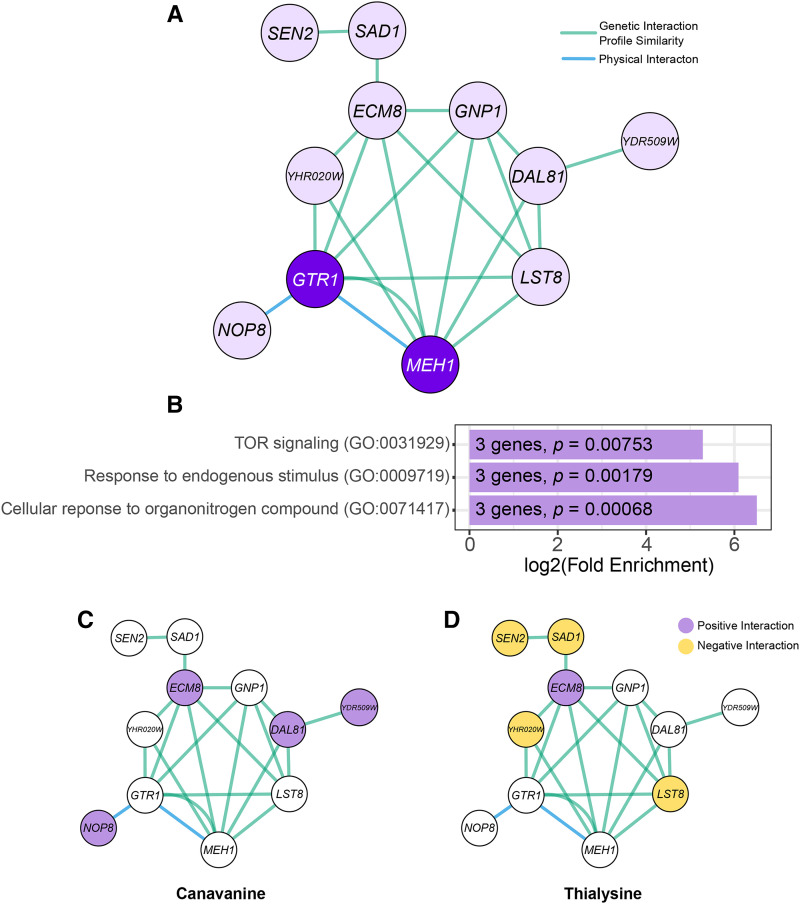
Genes that have positive chemical-genetic interactions with AZC. (A) Network of genes with positive chemical-genetic interactions with AZC. Genetic and physical interaction networks were generated using GeneMANIA. Nodes represent gene/proteins, green edges represent genes that have similar genetic interaction profiles (Costanzo *et al.* 2010, 2016) and blue edges represent proteins that physically interact. Dark purple indicates genes that are part of the EGO complex. (B) Significantly enriched GO biological processes were determined from the set of genes that have positive chemical-genetic interactions. The number of genes corresponding to each GO term and their *P*-values are labeled on each bar. (C) Genes with a chemical-genetic interaction with canavanine are shown on the network of genes having positive chemical-genetic interactions with AZC. Chemical-genetic interactions with canavanine were determined by growth in liquid media. Positive chemical-genetic interactions with canavanine are colored purple. (D) Genes with chemical-genetic interactions with thialysine are shown on the network of genes having positive chemical-genetic interactions with AZC. Chemical-genetic interactions with thialysine were determined by growth in liquid media. Strains with a positive chemical-genetic interaction with thialysine toxicity are colored purple; strains with a negative chemical-genetic interaction are colored yellow.

The 12 validated strains that had positive chemical-genetic interactions with AZC corresponded to deletion or mutation of 11 genes (Figure 7A; Supplemental File 3). GO analysis identified enrichment of genes involved in the cellular response to nitrogen compounds as well as TOR signaling (Figure 7B).

Three of the genes with positive chemical-genetic interactions with AZC function in AZC import. *GNP1*, a high affinity glutamine permease (Zhu *et al.* 1996), and *YDR509W*, a gene which overlaps with the 5′ coding sequence of *GNP1*, were both identified. Andréasson *et al.* (2004) demonstrated that Gnp1 is one of the permeases that imports AZC, and increased Gnp1 at the plasma membrane enhances AZC toxicity (Nanyan *et al.* 2019). In addition, AZC toxicity was suppressed by deleting *DAL81*. *DAL81* is required for *GNP1* expression, through the *S*sy1-*P*tr3-*S*sy5 (SPS) sensing pathway of extracellular amino acids (Boban and Ljungdahl 2007).

We tested if the twelve alleles with positive chemical-genetic interactions with AZC more generally suppress mis-incorporation using thialysine and canavanine (Figure 7C and 7D). Only deleting *ECM8* suppressed toxicity resulting from all three non-proteinogenic amino acids. *ECM8* is an uncharacterized gene whose deletion results in resistance to the aminoglycoside hygromycin b, an inhibitor of translation (Lussier *et al.* 1997). It is possible that deleting *ECM8* suppresses mis-made proteins by impacting translation levels. Interestingly, deletions of *YDR509W* and *DAL81*, described above to be involved in AZC uptake, also suppressed canavanine sensitivity suggesting these genes might have a broader role in the uptake of amino acid analogs.

Positive chemical-genetic interactions with genes involved in the TOR pathway is more specific since *GTR1**, **MEH1* and *LST8* only had positive chemical-genetic interactions with AZC. Both Gtr1 and Meh1 are part of the EGO complex which regulates sorting of the general amino acid permease Gap1 between the endosome and plasma membrane (Gao and Kaiser 2006). Gtr1 also activates TORC1 in response to amino acid stimulation (Panchaud *et al.* 2013). Downstream of TOR activation, Lst8 inhibits transcription factors that activate enzymes responsible for glutamate and glutamine biosynthesis (Chen and Kaiser 2003). Decreasing either TOR activation or downstream Lst8 activation increases glutamate and glutamine synthesis, which triggers targeting of a variety of amino acid permeases to the vacuole. Roberg *et al.* (1997) found that cells with the *lst8**-1* mutation are resistant to AZC, but sensitive to thialysine. Similarly, we observed that both *lst8**-6* and *lst8**-15* had negative chemical-genetic interactions with thialysine, supporting the idea that Lst8 regulates a specific set of amino acid permeases.

The temperature sensitive allele of *YHR020W*, the prolyl-tRNA synthetase, only had a positive chemical-genetic interaction with AZC and likely decreases the amount of AZC charged onto tRNAs. We observed a negative chemical-genetic interaction between this allele and thialysine, likely reflecting the combined toxicity of decreasing charged tRNA^Pro^ and mis-incorporating thialysine at lysine codons. It is less clear how the other genes with positive chemical-genetic interactions with AZC function. It should be noted two strains, *nop8**-101* and *sad1**-1*, had decreased fitness and that AZC could be suppressing the slow growth caused by the mutation, rather than the mutation suppressing AZC toxicity. Sen2 and Sad1, which only had positive chemical-genetic interaction scores with AZC, are involved in the splicing of tRNAs and mRNA, respectively (Winey and Culbertson 1988; Lygerou *et al.* 1999). Interestingly, we observed negative chemical-genetic interactions of *sen2**-1* and *sad1**-1* with thialysine, indicating that the interaction between these alleles and AZC is not the same for all types of proteotoxic stress. Nop8 is a nucleolar protein required for 60S ribosomal subunit biogenesis and physically interacts with Gtr1 (Zanchin and Goldfarb 1999; Todaka *et al.* 2005). The temperature sensitive *nop8**-101* allele also had a positive chemical-genetic interaction with canavanine. Nop8 may regulate Gtr1 and act through the TOR pathway discussed above for AZC or may play a more general role in controlling amino acid uptake.

## Conclusions

Using a chemical-genetic analysis we identified genes with positive and negative chemical-genetic interactions with AZC. Many of the genes with negative chemical-genetic interactions have roles in protein quality control and are involved in the proteasome, suggesting this is the main way cells degrade mistranslated proteins. We also identified genes involved in actin organization, endocytosis and vacuole sorting suggesting a possible role for the recycling of mis-made plasma membrane proteins in preventing toxicity due to mis-incorporation. These same processes are also required for cells to cope with two other non-proteinogenic amino acids, canavanine and thialysine and serine at proline mistranslation caused by a tRNA variant.

Mistranslated proteins, either with non-proteinogenic amino acids through genetic code expansion applications or with canonical amino acids using tRNA and aminoacyl-tRNA synthetase variants, have applications in synthetic biology (Bacher *et al.* 2005; Wang and Pan 2015; Reynolds *et al.* 2017; reviewed in O’Donoghue *et al.* 2013). Incorporating non-proteinogenic amino acids with novel side chains can expand functionality or specificity of a protein. Mistranslation with canonical amino acids yields “statistical proteins”, which are pools of molecules made from the same genetic template but with slightly different amino acid compositions and as such can broaden the function of a protein. Both situations result in proteotoxic stress by globally producing mis-made proteins that must be managed by the cell. Our screen has identified potential genes and pathways that carry out this function. As we have shown by overexpressing genes with negative chemical-genetic interactions, it is possible to modulate these genes and pathways to create strains more tolerate to mis-made protein.
